# Global and local identities on the balance scale: Predicting transformational leadership and effectiveness in multicultural teams

**DOI:** 10.1371/journal.pone.0254656

**Published:** 2021-07-14

**Authors:** Alon Lisak, Raveh Harush

**Affiliations:** 1 Department of Management, Ben-Gurion University of the Negev, Beersheva, Israel; 2 The Graduate School of Business Administration, Bar-Ilan University, Ramat Gan, Israel; Jacobs University Bremen, GERMANY

## Abstract

The performance of multicultural teams depends, to a great extent, on the effectiveness of their leaders. Transformational leadership is thought to be effective across organizational contexts and national cultures; yet we know little about what shapes these leadership behaviors. This study argues that leaders’ social identity configurations influence their transformational leadership behaviors and leadership effectiveness in multicultural settings. Building upon the global acculturation model, we test the effects of four identity configurations, based on the relative strength and balance of identification with the global and local cultures. We suggest that multicultural team leaders with balanced identity configurations, either glocal (high global, high local) or marginal (low global, low local), demonstrate more transformational leadership and consequently are more effective than leaders with unbalanced (dominant global or dominant local) configurations. Data were collected from 298 MBA students who worked on a four-week project in 77 multicultural teams. We used polynomial regression to capture how the discrepancy between the global and local components of leaders’ identity configurations affects transformational leadership behaviors and effectiveness. The results generally support the theoretical model, showing that the most transformational and effective leaders are those with balanced identity configurations. Theoretical and practical implications of the findings are discussed.

## Introduction

The growing presence of global organizations has led to the formation of multicultural teams consisting of culturally diverse members who operate across geographically dispersed zones [1]. Global organizations form multicultural teams in order to employ talented professionals worldwide, exploiting their diverse knowledge, skills, and perspectives [2,3]. Yet culturally diverse team dynamics frequently result in disagreements and mistrust as a consequence of disruptive social categorization processes derived from socio-cultural background and identity differences [4–6]. Effective leadership can minimize process losses and allow firms to capitalize on the potential of multicultural teams [7], something that is vital for organizational success [2,8]. However, despite the important potential contribution of effective leadership to multicultural team effectiveness, empirical and theoretical research on factors that lead to leadership effectiveness in this context is scarce [9,10].

To identify factors and behaviors that enhance leadership effectiveness in multicultural teams, this study explores transformational leadership. Transformational leadership is defined as leaders’ behaviors that influences followers by broadening and elevating their goals and providing them with confidence to perform beyond expectations specified in an implicit or explicit exchange agreement [11]. There are several reasons to assume that these behaviors are particularly likely to be effective in multicultural teams. First, the transformational leadership style is universally endorsed by both leaders and followers, across organizational contexts and national cultures [12–15]. Second, the literature consistently finds transformational leadership behaviors to be effective in diverse organizational and national contexts, as seen in several meta-analyses [16–18]. And third, some initial empirical findings suggest that transformational leadership behaviors can be effective in the multicultural context [7,9,10].

The present paper approaches the question from a slightly different angle. Additionally to probing the effectiveness of transformational leadership directly, we ask how the qualities that give rise to transformational leadership might help leaders be effective in their roles. Put differently, understanding the antecedents of transformational leadership behaviors in a multicultural context may help us identify the characteristics that promote successful leadership in such settings.

To narrow the scope of our investigation further, we explore whether and how leaders’ socio-cultural identities affect their transformational behaviors in a multicultural context. Socio-cultural identities are social identities that refers to “a broad range of beliefs and behaviors that one shares with members of one’s community” [19, p. 190], and that are known as drivers for individual adaptiveness and effectiveness in multicultural contexts [20–23]. People’s social identities are among the most substantial components of their self-concept, answering questions–like “Who am I?” and “How should I behave?”–that directly influence the individual’s actions [24,25]. Lord and Hall [26] argued that leaders’ social identities direct and shape their leadership behaviors in several ways: by providing an important structure around which relevant knowledge can be organized; by serving as a source of motivational forces driving leaders’ development; and by providing access to self-concept components that can be used to understand and motivate followers. Similarly, self-concept–based leadership theories suggest that charismatic leaders (as transformational leaders) reflect their social identities in their behaviors in a way that fosters a collective identity in their followers [27,28], as well as themselves [29]. Research findings on multicultural team contexts suggest that relevant socio-cultural identities such as global identity, can help leaders to be more effective [30,31].

To explore the relationship between leaders’ socio-cultural identity configurations and their transformational leadership behaviors, we rely on the global acculturation model [32], and particularly on a revised form of the model put forth in [33]. The global acculturation model “aims to explain individuals’ adaptation to the global environment by considering the relative strength of their local and global identities” [33, p. 1395], where *local identity* reflects an individual’s sense of belonging to the local–national culture, and *global identity* reflects a sense of belonging to a global, multicultural community [34,35]. The original global acculturation model [32], like other acculturation models (e.g., [36]), addresses the relationship between individuals’ identity configurations–i.e., the relative strength of their salient socio-cultural identities–and their ability to adapt to and function effectively within a particular cultural context. These general acculturation models suggest that individuals who adopt a bicultural identity configuration (strong identification with two cultures) are most effective, followed by a dominant cultural identity (strong identification with one culture). Low identification with both cultures, known as marginalization, is considered in these models an inferior acculturation strategy, leading to poor adaptation, weak social connections, and high psychological stress [21,22]. However, research findings on identity configurations and effectiveness in multicultural contexts have revealed a different pattern, where marginal individuals display higher effectiveness and adjustment than individuals with a dominant cultural configuration, although not as high as bicultural individuals [20,23]. To address this theoretical tension, Harush, Lisak, and Erez [33] introduced a conceptual development of the global acculturation model for settings where individuals from diverse national cultures operate in a shared global context. They claimed that the balance, or symmetry, between an individual’s local identity and global identity is a meaningful predictor of adaptive responses. The derived propositions are that individuals with glocal (high global, high local) or marginal (low global, low local) identities will respond to mixed cultural stimuli in a more inclusionary (or integrative) and less exclusionary way than individuals with unbalanced identities (who predominantly identify with one culture, global or local) [37].

However, to lead effectively in the multicultural team context, leaders need to not only react, but also to behave proactively [11,38]. Therefore, in this study, we further develop the global acculturation model by integrating ideas from social identity complexity theory–the study of how individuals represent to themselves the relationship between their different social identities [39]. We argue that a more complex relationship between coexisting social identities, in which the individual relates to multiple identities in a balanced way, allows leaders in multicultural contexts to proactively demonstrate more transformational leadership behaviors, making them more effective.

In exploring the relationship between leaders’ socio-cultural identity configurations, their transformational leadership behaviors, and their effectiveness in the multicultural team context, we make contributions to both the leadership and global acculturation literatures. First, this study contributes to transformational leadership literature by testing the claim of transformational leadership universalism in the multicultural context and identifying how leaders’ socio-cultural identities configuration contribute to their ability to perform transformational behaviors. We suggest that in a complex social environment, when members from multiple social groups are present simultaneously, it is more than one identity that matters, and it is leaders’ social identity configurations that may explain their transformational leadership behaviors and effectiveness. Thus, while this study explores a specific context, its theoretical and empirical contributions are broadly relevant to the literature on self-identities as a vital force shaping transformational and charismatic leadership behaviors [26,27].

Second, we conceptually and empirically link work on global acculturation and identity complexity [39–41] to explain why leaders with balanced identity configurations, even at low levels of identification, are more effective in the multicultural context than those with a dominant identity.

## Literature review and hypothesis development

### Transformational leadership and the multicultural team context

Transformational leaders inspire followers to perform at a higher level and to suspend their self-interest in favor of the collective good. They do this through four integrated leadership qualities, each expressed through different (though overlapping) sets of behaviors [42]. The first of these, *idealized influence*, refers to how transformational leaders exert influence by following their principles, serving as role models, and instilling a strong sense of purpose and collective mission in their teams [12,43,44]. The second, *inspirational motivation*, is an ability to motivate and inspire followers to find purpose in their work [45]. Both of those qualities are associated with charismatic leadership. The third quality is *individualized consideration*, or an ability to pay attention to followers’ unique needs [44,46,47]. Finally, the fourth is *intellectual stimulation–*encouraging followers to question assumptions and find solutions to problems [44]. The transformational leadership style has a stronger relationship with performance than transactional leadership, supporting the claim that charismatic and emotionally driven leadership behaviors lead to better performance than leadership behaviors which rely on an exchange relationship with followers [42,44,48,49].

Although transformational leadership is among the most explored areas in recent leadership research [50], the empirical study of transformational leadership in the multicultural context remains limited [10]. Yet, existing findings do suggest that transformational leadership may be effective in multicultural teams. For example, Kearney and Gebert [7] found that leaders’ transformational behaviors facilitated members’ team identification and task-relevant information elaboration, which led to higher team performance in diverse teams. Exploring leaders’ factors that enable transformational behaviors can contribute to our understanding of antecedents for this leadership style and consequently for leadership effectiveness in the multicultural team context.

A driving force for how individuals behave in diverse cultural environments are those individuals’ social identities of belonging to a particular community [35,36,51,52]. Social identity is “part of an individual’s self-concept which derives from his [sic] knowledge of his [sic] membership of a social group (or groups) together with the value and emotional significance attached to that membership” [53, p. 63]. Leaders’ social identities have been found to directly influence their role perception in the social environment [24,25] and their collective transformational behaviors [29]. Nevertheless, the available research on the possible contribution of leaders’ social identities to their transformational behaviors is limited, and "follower identity has received disproportionately more attention than leader identity” [29, p. 1262]. In the current study, we focus on aspects of leaders’ social identities that are particularly relevant to the multicultural context, and that are likely to influence leaders’ transformational behaviors, and consequently, their effectiveness. These are their local and global identities.

### Local and global identities and the acculturation perspective

Individuals’ attitudes and behaviors vis-à-vis their sociocultural context are shaped by their social identities [54]. Researchers have used acculturation models to explain the ease with which people adapt to and succeed in new cultural contexts, by assessing the strength of both the home (original) and host (new) cultural identity (e.g., [21,22]). Drawing on such models, Shokef and Erez’s global acculturation model [32] seeks to explain individuals’ ability to adapt and thrive in the multicultural context, based on the relative weight of their local and global cultural identities. In that model, an individual’s original cultural identity is represented by the concept of local identity, a cultural identity that reflects a sense of belonging to a particular national cultural group, with its shared meaning system, values, and symbols (e.g., language) [55]. Individuals who strongly identify with the local–national culture tend to view their co-nationals as an important ingroup that serves as a source of security [56]. At the same time, alongside their local identity, individuals who operate in global contexts can also develop a global identity [55]. Like other social identities, a global identity reflects an individual’s sense of belonging to and identification with a particular social group [54]–in this case, the global work community in general, and global units such as multicultural teams in particular [5,23,35]. Individuals with high levels of global identity are interested in overcoming cultural barriers and maintaining positive relationships with others who operate in the global context, and who share a common global culture [30,34,57]. That said, while members of culturally diverse teams may regard their global identity as creating a shared ingroup, this global identity should not be conceived as a superordinate group containing nested national subgroups. Rather, the global group and local cultural groups should be seen as cross-cutting categories [57].

To explain individual effectiveness in a global work context, Shokef and Erez [32] describe four identity configurations based on the relative strength of the global and local identities: glocal type (high global–high local), marginal type (low global–low local), global type (high global–low local), and local type (low global–high local). Shokef and Erez [32,58] argue that individuals who conform to the glocal identity type are the most effective in global contexts because their high identification with both cultures means they are able to integrate perspectives related to both the local culture and the global culture of their units. This claim builds on the logic of previous national-level acculturation models, which emphasize the advantages of identity integration and consider marginalization to be the least effective acculturation strategy [21,22,59,60]. However, we argue that the same logic does not apply to a multicultural context in which individuals from multiple “home” (local) cultures work in a shared global cultural context, simultaneously experiencing local and global cultures [58]. In the present study we present an alternative logic based on the balance between identities as key to an effective strategy–and, as we shall later see, to effective leadership–in the multicultural team context.

### Identity configurations, balance, and effectiveness in the multicultural environment

Findings on adaptiveness and effectiveness in cross-cultural contexts show that bicultural individuals, who identify strongly with two national (local) cultures, tend to score higher in measures of creativity, effectiveness, cultural intelligence, and adjustment compared with those who identify disproportionately with either the home or host culture (i.e., who are separated or assimilated, respectively) [20,61–63]. Unexpectedly, marginal individuals–whose identification with both the home and host cultures is equally low–also tend to score higher in creativity, effectiveness, cultural intelligence, and adjustment compared with assimilated or separated individuals, although not to the extent of biculturals [20,23,63]. This general pattern of findings poses a theoretical challenge to acculturation models that a priori separate the discussion of marginalization from the general discussion of balance, or that do not discuss marginalization at all [23,32,36]. For example, Tadmor and Tetlock [36] chose not to include marginalization in their acculturation complexity model, on the grounds that individuals who adopt this strategy are less likely to feel accountable to any cultural group. Notably, as one possible explanation of their results, Tadmor et al. [20] suggested that marginal individuals, like bicultural individuals, may also have a balanced identity structure, though one characterized by weaker identification, and that this balance leads to higher identity complexity, cognitive complexity, and effectiveness compared to unbalanced identity configurations.

**Fig 1:**
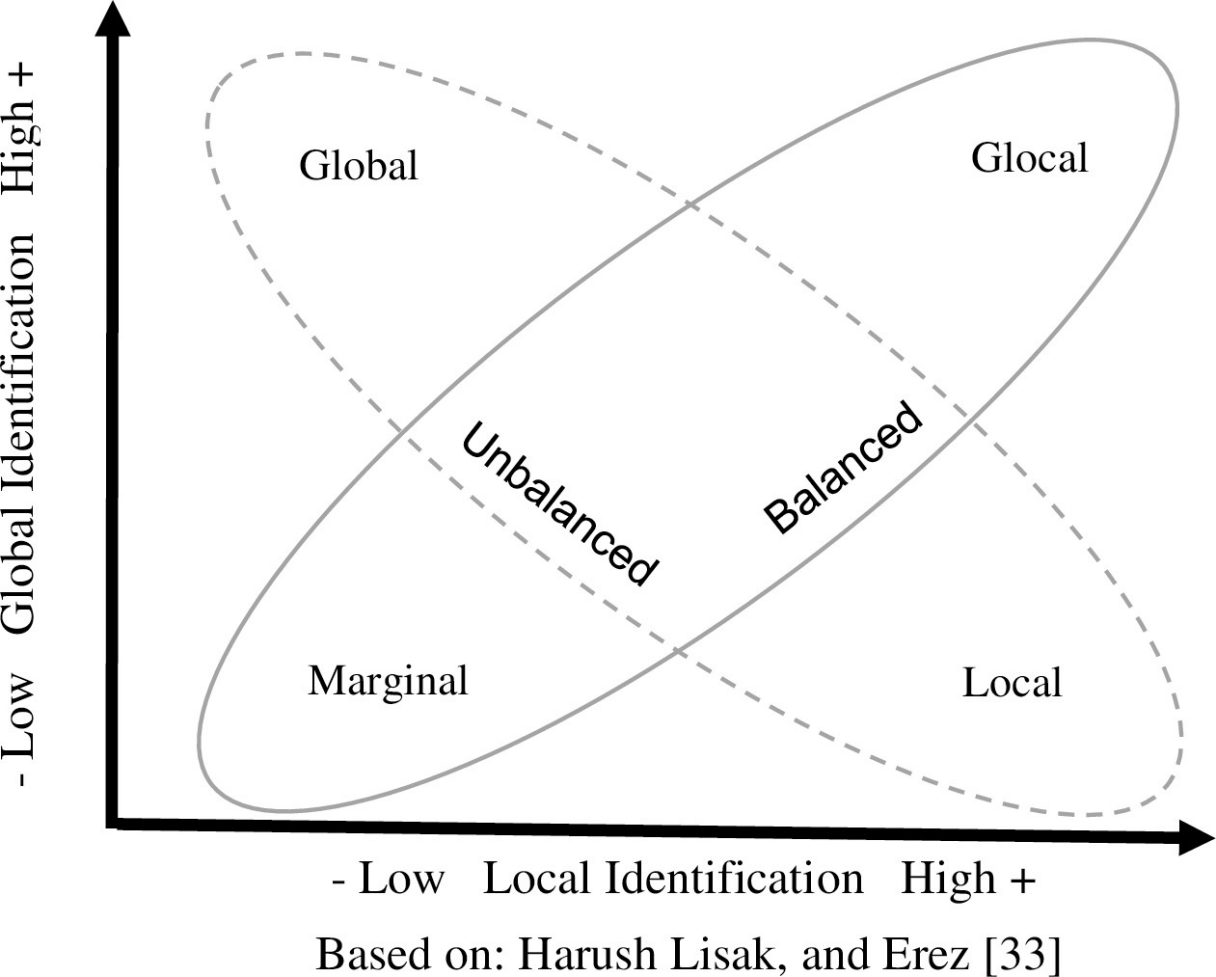
Extended global acculturation model.

In the multicultural context, Harush et al. [33] also suggested shifting the focus from identity strength per se to balance as a predictor of effectiveness. Accordingly, identity configurations characterized by symmetry between the global and local identities (the glocal and marginal types) are classified as balanced, while configurations characterized by one dominant identity (the global and local types) are classified as unbalanced (see Fig 1). Yet this shift in focus to the balance between two identities still requires an explanation of the underlying mechanisms by which balance, or the lack of it, affects how well leaders thrive in a multicultural context. Towards this end, we conceptually develop previous suggestions [20,33] linking balanced configurations to identity complexity [39], and delineate how these configurations are related to effective cognitive, emotional, and behavioral tendencies in multicultural settings.

### Linking (un)balanced configurations to identity complexity

Social identity complexity is an individual’s subjective self-representation of the interrelationships among their multiple social identities [39]. In less complex representations, a dominant identity may suppress other identities–what Roccas and Brewer [39] call a dominance identity structure. In more complex identity representations, individuals acknowledge and relate to multiple identities simultaneously.

Considering identity configurations in the global context, individuals with unbalanced configurations identify primarily with either the global or local culture, to which the other identity (local or global) is subordinated. Individuals with a dominance identity structure classify people on this basis, categorizing those who share their dominant identity as ingroup members and those who do not as outgroup members. In denying or suppressing one identity, such a representation fosters a simplistic, monolithic view, bolstering the commitment to one identity over the other [39,40]. By contrast, individuals with balanced configurations (i.e., glocals and marginals) have no dominant identity [33], meaning they are high in identity complexity.

The social identity complexity perspective [39] attributes high complexity only to individuals who follow an integrative acculturation strategy and who identify strongly with both cultures. Like other theories, this theory also refrains from discussing the marginal acculturation strategy, in which both identities are relatively low in strength. However, we can find an alternative conceptualization in recent work by Vora and colleagues [64], who suggest that multiculturalism at the individual level is a function of three factors: knowledge of, identification with, and internalization of more than one culture. Using this conceptualization, we can argue that marginals may indeed be low in identification and internalization; but because they allow both their identities to remain in play (i.e., no identity either dominates or is suppressed), their knowledge about both identities may remain high. Thus, linking the work of Vora et al. [64] with social identity complexity theory, we may argue that a marginal social identity is indeed potentially complex. This idea aligns with previous findings suggesting that marginals manifest higher levels of both identity complexity and cognitive complexity than individuals who identify with only one culture [20].

### Multicultural team leaders’ identity balance and transformational leadership

Leadership research has found that transformational leadership behaviors are associated with both cognitive and emotional complexity. High levels of cognitive complexity (including attributional complexity -the capacity to discriminate between and to integrate dimensions of social judgment) enhance transformational leaders’ ability to serve as role models, to inspire, to provide intellectual stimulation, and to consider followers as individuals [45,65]. These qualities of transformational leadership are also positively related to emotional complexity–an ability to manage both one’s own emotions and those of others [66–69]. In the multicultural context, too, a positive relationship exists between identity complexity, cognitive complexity, and emotional complexity [70,71]. Lakshman [71] claimed that among bicultural leaders, as opposed to those from only one cultural background, high attributional and knowledge complexity leads to a more accurate and unbiased cultural perspective, and in consequence to more effective charismatic-transformational leadership in multicultural contexts. Fitzsimmons et al. [70] suggested that such an unbiased and knowledge-based cultural perspective also improves the effectiveness of global leaders with marginal identity configurations, who can use their knowledge about global and local cultures in a balanced fashion to promote their goals.

Conceptually linking balanced identity configurations to identity complexity complements this line of thought by explaining why leaders with balanced configurations might demonstrate more transformational leadership behaviors relative to leaders with unbalanced identity configurations. Cognitively, identity is related to perceptions of group membership and the categorization of self and others into social groups [72]. Higher levels of identity complexity enable a more reliable representation of the multicultural social context [40,71], and contribute to a recognition that followers may simultaneously be members of several different social groups [36,39,73]. That is, leaders with balanced–and complex–identity configurations are well-positioned to relate to their team on the basis of both their common global team identity, and team members’ unique local identities. The emotional component of social identity, for its part, captures the feeling of belonging, the emotional significance attached to group membership, the motivation to be concerned about the group’s welfare, and–importantly–bias in favor of the ingroup and against the outgroup [72]. Leaders with balanced configurations have equal attachment to both cultural groups and so should be less prone to bias, while leaders with unbalanced identity configurations are likely to be motivated to show greater concern for team members who identify with their preferred cultural group [40,41].

Based on the proposed logic, we suggest that leaders with balanced global–local identity configurations are more likely than leaders with unbalanced identity configurations to demonstrate transformational leadership behaviors. Leaders with balanced social identity configurations have a cognitive awareness of cultural complexity, and an emotional balance, that together reduce bias and enhance tolerance toward diverse members of the team. Therefore, they are likely to behave in ways that are more thoughtful, culturally responsive, and less biased than leaders with unbalanced identity configurations (individualized consideration). At the same time, these leaders understand the need to connect followers to a shared system of meaning, they inspire followers to work for the good of the team by following their principles, serving as role models, and instilling a sense of purpose (idealized influence and inspirational motivation) [74,75]. Finally, they guide the team in harnessing the benefits of members’ diverse perspectives to find optimal solutions to team challenges (intellectual stimulation) [7].

Conversely, multicultural team leaders with unbalanced identity configurations, who have one dominant identity and low identity complexity, are less likely to demonstrate transformational leadership behaviors. Such leaders that deny or suppress one of their identities, tend to adopt simplistic, monolithic views based on their dominant identity, and are disproportionately committed to the dominant identity. In particular, leaders with a global identity configuration may fail to acknowledge that team members belong to diverse local–national groups, or to value their diverse and unique perspectives. This will hinder their ability to manifest appropriate culture-sensitive individual consideration and to provide the intellectual stimulation needed to harness the benefits of cultural diversity for the collective good. Leaders with a local identity configuration, for their part, are likely to classify local cultures other than their own as outgroups, impeding their ability to manifest any of the components of transformational leadership: to treat each member with the appropriate individual consideration, to unify the team around a shared global meaning, to stimulate the group intellectually around a shared understanding, or to inspire and motivate followers.

Based on the foregoing, we expect that a balance between leaders’ global and local identities will be linked to high levels of transformational leadership in the multicultural team context, compared with an imbalance between these identities. Formally we hypothesize:

H1: *Multicultural team leaders with balanced identity configurations (glocal or marginal) will demonstrate higher levels of transformational leadership than leaders with dominant (i*.*e*., *imbalanced) identity configurations (global or local)*.

### Multicultural team leaders’ identity balance and leadership effectiveness

Findings in cross-cultural contexts have consistently revealed that individuals with balanced identity configurations (both biculturals and marginals) are better able to adapt and flourish in host cultures than individuals with unbalanced identity configurations [20,23,63]. In this study we extend this reasoning by proposing that team leaders with balanced identity configurations (glocal or marginal) will be more effective in multicultural settings than leaders with one dominant identity (global or local) because they demonstrate higher levels of transformational leadership behaviors. Transformational leadership behaviors promote team processes that facilitate individual and team performance, such as trust, communication, information elaboration, and team identification [7,76,77]. There is also a vast body of evidence linking transformational leadership with measures of effectiveness, such as individual-level and team performance [16,50].

Transformational leaders are thought to be effective in the multicultural team context because of their ability to promote shared (global) team goals and strengthen followers’ sense of belonging to the team while showing respect and giving voice to diverse cultural perspectives [8,39,78]. These capacities build strong leader–follower relationships [11,79] and lead to high scores on follower-reported measures of perceived leadership effectiveness [16,50].

To reiterate, transformational leadership is facilitated by and reflective of a balanced identity configuration. Therefore, we propose both that having a balanced social-cultural identity (glocal or marginal) will be positively related to leadership effectiveness in the context of multicultural teams; and that this relationship will be mediated by transformational leadership behaviors. Formally, we hypothesize:

H2: *Multicultural team leaders with balanced identity configurations (glocal or marginal) will be more effective than leaders with one dominant identity (global or local)*.

H3: *Transformational leadership behaviors of multicultural team leaders will mediate the relationship between their identity configurations and their leadership effectiveness*.

## Method

### Ethics approval

The first author collected the data during his stay at the Technion—Israel Institute of Technology. The Technion Helsinki Committee approved this study.

### Participants

The study sample comprised 298 MBA students representing 40 nationalities who were enrolled at eight universities worldwide (one each in Finland, Hong Kong, India, Israel, and Spain; three in the United States), and who participated in a multicultural team project. Of the participants, 36% were European (9% from Italy, 7% from Germany, and 20% from other European countries), 20% were from the Far East (with 16% being Chinese), 16% were Israeli, 14% were North American, 7% were Indian, and 7% were from other areas (e.g., Latin America and Central Asia). The average age was 27.35 years (SD = 5.70), and 64% were men. Most participants (69%) had previous work experience, and most participants (75%) reported that they had previously worked in multicultural teams. A precondition for participation in this study was a sufficient level of English proficiency for fluent intra-team communication (e.g., via e-mail, instant messaging/chats, and video calls). The mean self-reported level of English proficiency was 4.54 (SD = .70, 1–5 scale), and 80% of participants were enrolled in English-language MBA programs.

### Procedure

Participants were assigned to 77 virtual multicultural teams, of which 67 teams (87%) consisted of four members, and the remaining ten of three members. Participants were randomly assigned to the teams based on three criteria: that all members were from different countries, were of different nationalities, and attended different universities. Participants then worked on a four-week team project as part of the requirements of a cross-cultural management course. The project was a significant part of each participant’s final course grade (between 40% and 60%).

The study had four phases:

#### *Phase 1*: Pre-project

Before beginning the project, all participants filled out a web-based questionnaire assessing their global and local identities. We used these scores to assess the identity configurations of the leaders that were elected at the end of Phase 2.

#### *Phase 2*: Getting to know each other

During the first nine days of the project, team members engaged in group chats via instant messaging, and in daily e-mail exchanges. To help them become better acquainted, the teams were also asked to discuss a case study involving a personal moral dilemma. The teams then chose a country about which they were to write their final project together.

At the end of Phase 2, team members were asked to elect “the most suitable team member” to become their team leader and lead them through their assignment in Phase 3. Similar to the general sample, about two-thirds (67%) of the leaders were men, and their average age was 27.87 years (SD = 6.28). Also, 26% of the leaders were European, 23% were from the Far East (21% Chinese), 22% were Israeli, 17% were North American, 9% were Indian, and 3% were from other regions. The proportion of nominated leaders from English-speaking countries (17%) was close to the proportion of participants from such countries in the sample (15%), suggesting no bias in leadership emergence for native English speakers.

#### *Phase 3*: Team project

This phase, which lasted 19 days, consisted of the team assignment. The task was to develop guidelines for an expatriate assignment for a position in a country selected by the team (not the home country of any of the team members).

#### *Phase 4*: Project wrap-up

At the end of the project, members evaluated the extent to which their team leader exhibited transformational leadership behaviors, and how effective they were as leaders throughout the assignment.

### Measures

*Global identity* and *local identity* were measured by leaders’ self-reports, using the identity scales developed and validated by Shokef and Erez [32,55,58]. Both scales consisted of five items, with responses on a 7-point Likert-type scale (1 = not at all; 7 = very much). The global identity scale measured the individual’s sense of belonging to the global social group (e.g., “I see myself as part of the global international community”), while the local identity scale consisted of five items that measured the individual’s sense of belonging to the local–national group (e.g., “I define myself as an ____ (your nationality, e.g., American, Korean, etc.)”). Cronbach’s alpha was .92 and .91 for the global and local scales, respectively. To confirm that the global and local identity scales in fact represent two different factors, we conducted a confirmatory factor analysis (CFA). The two-factor model yielded a satisfactory fit (χ^2^_(33)_ = 134.24, *p* < .01; CFI = .92; TLI = .90; SRMR = .078), while an alternative one-factor model produced insufficient fit indices (χ^2^_(34)_ = 724.45, *p* < .01; CFI = .46; TLI = .26; SRMR = .240). Comparison of the χ^2^ values for the two models revealed significant differences between them (Δχ^2^_(1)_ = 590.21, *p* < .01), further supporting the two-factor model.

*Transformational leadership* of team leaders was evaluated by team members using 16 items from the Multifactor Leadership Questionnaire-5X short (MLQ [47]). Responses were given on a 5-point Likert-type scale (0 = not at all; 4 = frequently, if not always). These 16 items reflected the four subscales of transformational leadership (four items each): a) individualized consideration (sample item: “Treats me as an individual rather than just as a member of a group,” α = .75); b) intellectual stimulation (sample item: “Suggests new ways of looking at how to complete assignments,” α = .87); c) inspirational motivation (sample item: “Talks enthusiastically about what needs to be accomplished,” α = .84); and d) idealized influence (behavioral, sample item: “Emphasizes the importance of having a collective sense of mission,” α = .80). To justify using the total measure of transformational leadership, we took two steps. First, following previous studies which found theoretical and methodological justification for combining idealized influence and inspirational motivation to form one “charisma factor” [48,79,80], we combined these two subscales into one subscale (α = .90). Then, we conducted a second-order CFA analysis with these three subscales as first-order factors. Fit indices for the three first-order factors plus the second-order factor fell within an acceptable range (χ^2^ (101) = 259.29, p < .01; CFI = .92; TLI = .90; SRMR = .062), enabling us to use the total measure of transformational leadership (α = .94).

*Leadership effectiveness* was evaluated by team members using a leadership effectiveness scale modified for use in projects (based on [81]). The scale consisted of six items, with responses on a 5-point Likert-type scale (1 = strongly disagree; 5 = strongly agree). Sample item: “Our team leader succeeded in his/her role during the project” (α = .94).

To confirm that the transformational leadership and leadership effectiveness variables indeed represent different constructs, we conducted another CFA. The hypothesized two-factor model provided evidence of a satisfactory fit (χ^2^_(34)_ = 162.89, *p* < .01; CFI = .92; TLI = .90; SRMR = .066), while an alternative one-factor model demonstrated insufficient fit indices (χ^2^_(35)_ = 225.36, p < .01; CFI = .88; TLI = .84; SRMR = .077). A comparison of the χ^2^ values of the two models revealed significant differences between them (Δχ^2^_(1)_ = 62.7, *p* < .01). Therefore, we continued to examine these variables as two distinct constructs.

*Common Method Variance (CMV) test for transformational leadership and leadership effectiveness*. Both transformational leadership and leadership effectiveness were measured by team members, creating a risk of common method bias. To test for this possibility, we used a confirmatory factor analysis marker technique. Following Podsakoff [82,83], we used social desirability as a marker, as social desirability is theoretically uncorrelated with transformational leadership and leadership effectiveness. Specifically, we used five items from the Balanced Inventory of Desirable Responding (BIDR; [84]) to model the marker variable (sample item: "I never regret my decisions”; 1 = not true, 5 = very true; α = .70). To assess the possible influence of CMV, we then tested a series of models, following the procedure of [85,86]. The results are presented in S1 Appendix. They indicate that the presence of CMV does not appear to bias the relationships between transformational leadership and leadership effectiveness.

*Control variables*. We controlled for other leader characteristics that have been found to be related to leadership effectiveness in general and the global context in particular. Specifically, we controlled for leaders’ general self-efficacy using the eight-item scale of Chen et al. [87] (1 = strongly disagree, 5 = strongly agree; sample item: “I will be able to achieve most of the goals that I have set for myself”; α = .84). Additionally, we controlled for the leaders’ openness to experience, using five items from the International Personality Item Pool (IPIP) [88] (1 = very inaccurate, 5 = very accurate; sample item: “I am not interested in abstract ideas” (R); α = .84). Although the teams were virtual, about 40 percent of the leaders were physically located in host countries. Hence, we controlled for their location (host country/home country). We also controlled for the number of languages spoken by both leaders and team members, and for members’ global and local identities (using the same scales as for the leaders) [23]. Finally, we controlled for team demographics, and specifically age and gender [23], using age diversity in the team (as expressed by the SD) and the proportion of women in the team [89].

### Analytical strategy

In this study, we use polynomial regression with a response surface to explore our hypotheses. This method suggests a solution to the problems associated with using difference scores to analyze discrepancies in ratings [90–92], as it “has more explanatory potential than do difference scores or traditional moderated regression analyses and holds promise for applicability to a wide range of research questions” [93, p. 543]. Moreover, the method retains the independent effect of each component measure, exposing the unique contribution of each to the variance of the outcome, which helps overcome confounded effects. Finally, unlike linear regression, polynomial regression reveals non-linear changes along the lines, enabling a more nuanced view of the outcome [93,94].

**Fig 2:**
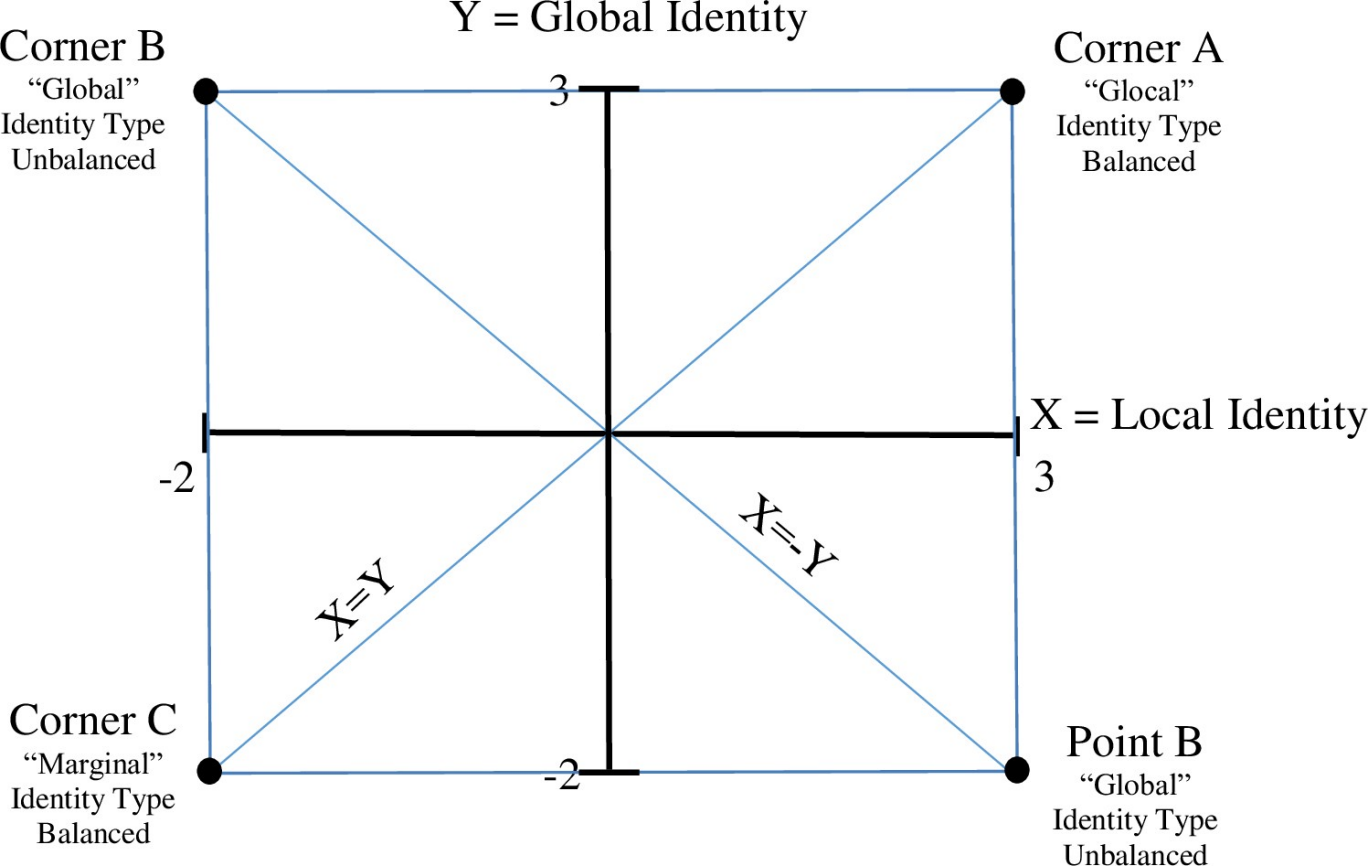
Schematic description of lines of interest between the four identity types.

Lee [63] noted that although the polynomial regression and response surface methodology is commonly used for testing congruence hypotheses, it is also applicable for examining the joint effects of dual cultural identity configurations, represented by four combinations of the extreme values of these identities, as located at the four “corners” of the response surface. In our study, the four corners of the surface represent the four configurations of global and local identities: Corner A represents the glocal (high global–high local) identity configuration; Corner B the global (high global–low local) configuration; Corner C the marginal (low global–low local) configuration; and Corner D the local (low global–high local) identity configuration (see Fig 2). The four lines along the edges of the response surface in Fig 2 connect balanced identity types with unbalanced identity types, while the diagonal lines connect the two balanced identity types (the fit line, corners C–A) and the two unbalanced types (the misfit line, corners B–D). The relationships captured by the lines at the edges were used to test our hypotheses. The relationships captured by the diagonal lines were not central to our hypotheses; nonetheless, we report the results of those analyses below.

Prior to the analyses, the measures of global and local identities were scale-centered by subtracting the midpoint of the scale (i.e., subtracting 4) [90]. Additionally, as no scores for the leader’s global or local identity variable were less than 2, we explored the response surface for these variables within the range of 2 to 7 (or -2 to 3 after subtracting the midpoint of the scale), to avoid extrapolations in our analysis [95]. The need to avoid extrapolation is essential when using polynomial regression, as this method is extremely sensitive to possible bias when working beyond the range of the data [96–98].

To test our research model, we conducted a mediated multi-level polynomial regression procedure (based on the procedures described by Zhang et al. [99]) and response surface modeling [92] using the SAS 9.4 MIXED procedure. Specifically, we employed a cross-level polynomial regression (i.e., a combination of hierarchical linear modeling and polynomial regression [100–102]), examining the relationships between leaders’ global and local identities (a team-level variable) and their followers’ perceptions of their transformational leadership and effectiveness (individual-level variables). This cross-level approach enables us to explain more variance at the individual level than would be explained by single team-level formulations [102]. Moreover, cross-level polynomial regressions enable us to account for non-independence among team members working with the same leader by applying random intercept polynomial models (indeed, scores for transformational leadership and leadership effectiveness from members nested within the same team were correlated).

The dependent variables–transformational leadership (H1) and leadership effectiveness (H2)–were regressed on five polynomial terms: leader’s global identity, leader’s local identity, their squares, and their product. Next, to test the nature of the relationships between balanced and unbalanced identity types, we conducted additional analyses. First, based on the procedure developed by Lee and Antonakis [103], we tested the equality between the predicted values of the criteria (transformational leadership/ leadership effectiveness) at the four corner points to examine whether the predicted values at the “balanced” corners (glocal and marginal) were significantly higher than the values at the “unbalanced” corners (global and local). We used the ESTIMATE statement of the SAS MIXED procedure to estimate the differences between the values of both transformational leadership and leadership effectiveness in different corners of the response surface, and conducted an approximate t-test for the significance of these differences.

Second, we tested the curvatures of the interest lines that connected the four corner points (i.e., the balanced and unbalanced identity types) along the edges of the response surface. For this purpose, we developed equations to test the curvature significance of these four lines based on work by Cohen et al. [104] (see S2 Appendix for an explanation). Next, for lines with a significant curvature, we also tested the tangent slopes along these lines (based on the procedure developed by Lee and Antonakis [103]). Each tangent slope is equal to the slope of the tangent line drawn at a specific point along an interest line. This enabled us to examine the functional form of the interest lines with greater precision.

To test our third hypothesis, according to which transformational leadership mediates the joint effect of leaders’ global and local identities on their perceived leadership effectiveness, we probe the indirect effect, using the block variable method [99,105]. Accordingly, to obtain a single coefficient that represented the joint effect of the five polynomial terms (leader’s global identity, leader’s local identity, their squares, and their product), the five terms were combined into a block variable that was their weighted linear composite. The joint indirect effect of global and local identities on perceived leadership effectiveness, through transformational leadership, was calculated as the product of (a) the standardized regression coefficient of the block variable on transformational leadership, and (b) the standardized regression coefficients of transformational leadership on leadership effectiveness, in the presence of the direct effects of leaders’ global and local identities. We used the Monte Carlo confidence interval (MCCI) method [106] to create 95% confidence intervals that assessed the indirect effect with 20,000 replications, because this method can be applied while accommodating the interdependence induced by the clustering of our data [107].

## Results

Table 1 presents the means, standard deviations, and correlations between the research variables.

**Table 1:** Descriptive statistics and correlations for study variables.

	Variable	M	SD	1	2	3	4	5	6	7	8	9	10	11	12
1.	General self-efficacy (leader)	4.09	0.44	-											
2.	Openness (leader)	3.65	0.76	.22**	-										
3.	No. of languages (leader)	2.58	0.71	-.03	-.05	-									
4.	Leader in host/ home country ^a^	0.43	.050	-.12	-.09	.43**	-								
5.	Global identity (members)	5.07	1.04	-.10	-.02	-.05	-.08	-							
6.	Local identity (members)	5.10	1.22	-.02	-.11	.04	-.02	-.01	-						
7.	No. of languages (members)	2.55	0.73	-.02	.06	-.12	-.05	.33**	-.12	-					
8.	Gender proportion^b^	0.35	0.19	.25**	-.07	.18**	.02	.10	.10	-.06	-				
9.	Age diversity ^c^	5.38	2.63	-.03	-.01	-.23**	-.09	-.02	.02	-.08	-.29**	-			
10.	Global identity	5.05	0.94	.26**	.29**	.20**	-.07	-.04	-.01	-.10	.16*	.19**	-		
11.	Local identity	5.23	1.08	.22**	.08	-.15*	-.17*	-.05	-.04	-.08	.01	.11	.26**	-	
12.	Transformational leadership	2.32	0.80	.05	-.07	.21**	.05	.07	.10	-.05	-.05	.02	.06	.10	-
13.	Leadership effectiveness	3.70	0.89	.14*	-.09	.25**	.12	.06	.06	-.03	.01	-.06	.04	.06	.71** ^d^

^a^ Leader location: 0-Local country, 1-Host country

^b^ Gender proportion of Women: 0-Female, 1-Male

^c^ Age diversity by SD

^d^ Although the correlation with transformational leadership may seem high, the standardized partial correlation, when controlling for the rest of the quadratic polynomial terms and the control variables, is .65.

N = 298 (221 members, 77 leaders).

* *p* < .05

** *p* < .01.

To test the joint effect of the leaders’ global and local identities on both transformational leadership (H1) and leadership effectiveness (H2), we conducted a polynomial regression analysis [93]. Transformational leadership and leadership effectiveness were regressed (separately) on leaders’ global identity and local identity in Step 1. The interaction between global and local identities, the square of global identity, and the square of local identity were added in Step 2. A significant *F* test for the three quadratic terms in Step 2, for both transformational leadership (F = 4.90, *p* < .01) and leadership effectiveness (F = 4.25, *p* < .01), indicated a non-linear relationship between identity and these dependent variables [94]. This supported further examination of the quadratic relations (see Table 2).

**Table 2:** Results of polynomial regression.

Variable	Model 1: Transformational leadership		Model 2: Leadership effectiveness		
	Step 1	Step 2	Step 1	Step 2	Step 3
Constant	2.08* (0.20)	2.46** (.19)	3.70** (.23)	3.83** (.22)	2.08** (.20)
General self-efficacy (leader)	0.23 (.17)	0.15 (.16)	0.48* (.20)	0.40* (0.19)	0.29* (.12)
Openness (leader)	-0.09 (.09)	-0.03 (0.09)	-0.13 (.11)	-0.08 (0.11)	-0.06 (.07)
No. of languages (leader)	0.29 * (.12)	0.33**(.11)	0.32** (.14)	0.37** (0.13)	0.14 (.09)
Leader in host/ home country	-0.05 (.15)	-0.10 (.15)	0.07 (.18)	0.04 (0.17)	0.10 (.11)
Global identity (members)	0.08 (.05)	0.07 (.05)	0.09^†^ (.05)	0.08 (.05)	0.03 (.04)
Local identity (members)	0.07 (.04)	0.07 (.05)	0.05 (.04)	0.06 (.04)	0.01 (.03)
No. of languages (members)	-0.02 (.07)	-0.03 (0.09)	0.02 (.07)	0.01 (.07)	0.02 (.06)
Gender	-0.63 (.40)	-0.56 (.37)	-0.64 (.46)	-0.55 (.44)	-0.16 (.29)
Age diversity	0.01 (.02)	0.03 (.03)	-0.01 (.03)	0.01 (.03)	-0.01 (.02)
Global identity	0.02 (.09)	-0.30* (.13)	-0.03 (.10)	-0.38* (.15)	-0.16 (.10)
Local identity	0.02 (.07)	0.12 (.14)	0.02 (.08)	0.07 (.16)	-0.01 (.10)
Global identity X Local identity		0.17* (0.08)		0.22* (.10)	0.10 (.06)
Global identity squared		0.08 (.06)		0.05 (.08)	0.01 (.05)
Local identity squared		-0.11* (.05)		-0.12* (.06)	-0.04 (.04)
Transformational leadership					0.70** (0.06)
F for the three quadratic terms		4.90**		4.25**	1.23

*Note*: Unstandardized coefficients are reported. Standard errors appear in parentheses.

*p < .05

** *p* < .01.

**Fig 3:**
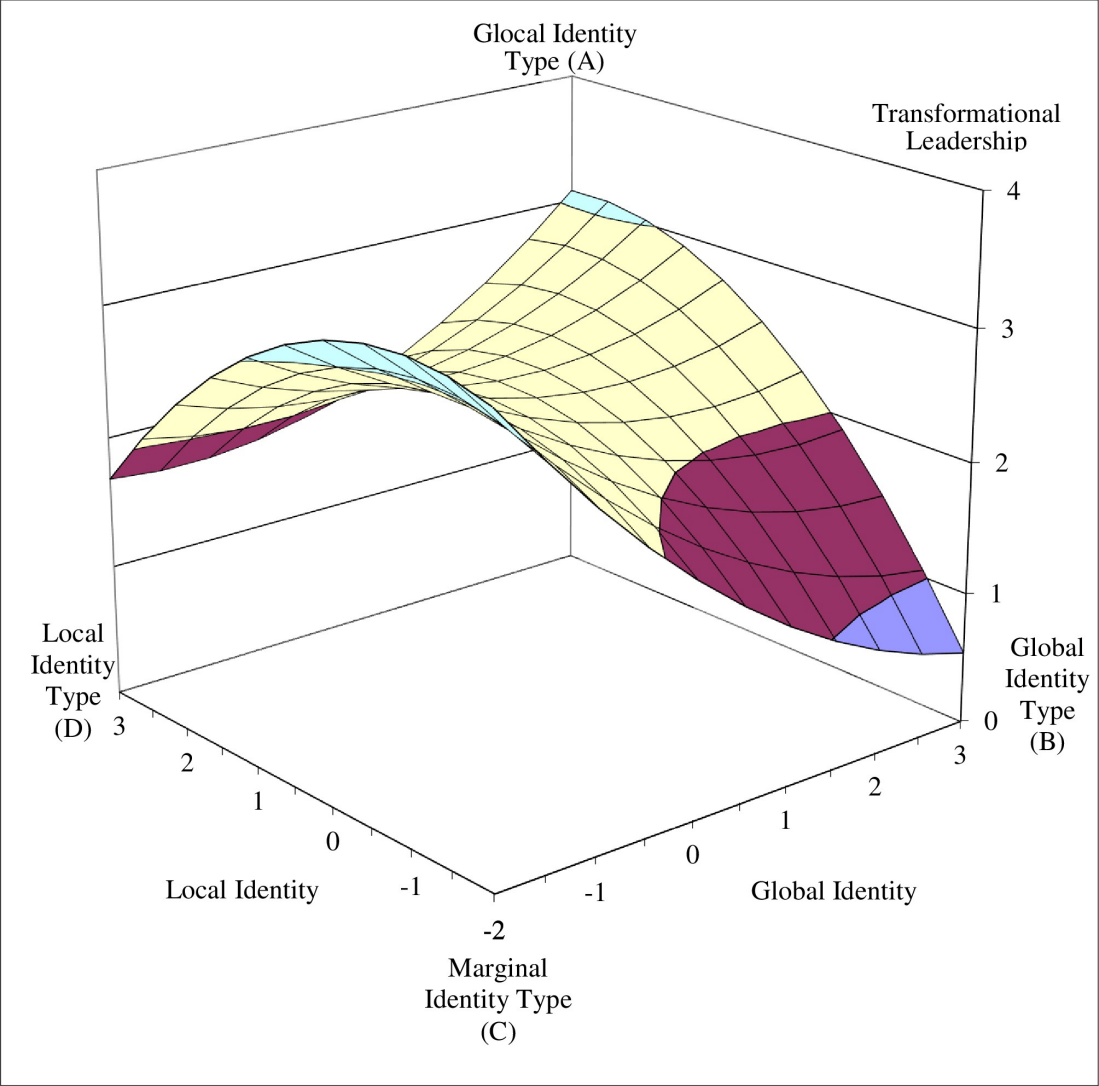
The four identity types as predictors of transformational leadership.

**Fig 4:**
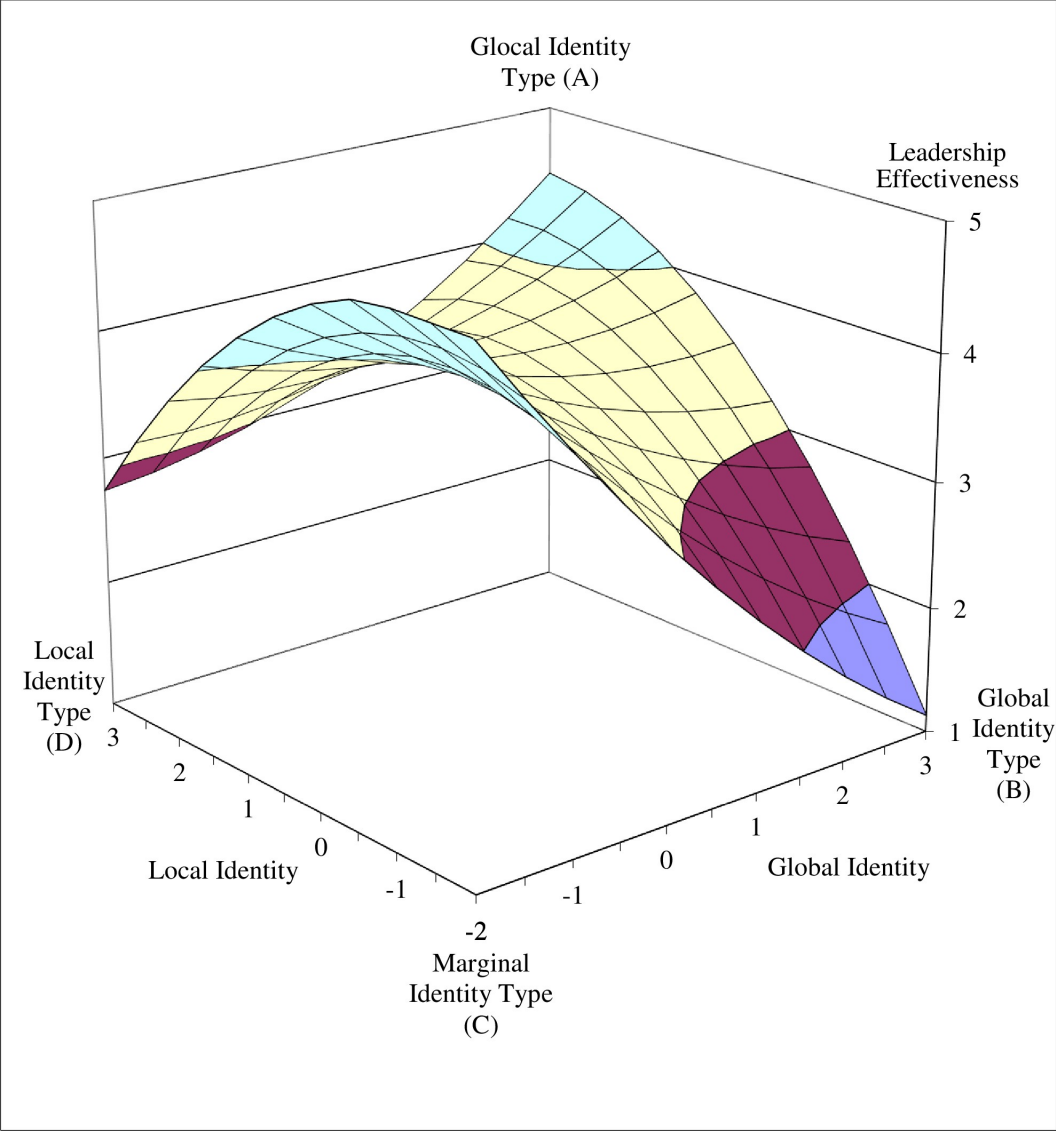
The four identity types as predictors of leadership effectiveness.

To better interpret the nature of the quadratic polynomial regression models, we plotted the response surfaces of the estimated models for transformational leadership and leadership effectiveness (see Figs 3 and 4). To explore Hypotheses 1 and 2, we first tested whether the predicted values of the four corner points on the response surfaces differed (Table 3).

**Table 3:** Testing the equality between predicted values on the response surfaces.

	Transformational leadership	Leadership effectiveness
	Predicted value at specific point	
Points on the response surface		
A (Glocal)	3.08	4.33
B (Global)	0.54	1.22
C (Marginal)	3.37	5.09
D (Local)	1.68	2.61
	Differences between predicted values	
Along the edges		
A (Glocal) vs. B (Global)	2.54*	3.12**
A (Glocal) vs. D (Local)	1.40^†^	1.71^†^
C (Marginal) vs. B (Global)	2.83 *	3.88**
C (Marginal) vs. D (Local)	1.69	2.48^†^
Along diagonal lines		
C (Marginal) vs. A (Glocal)	0.29	0.76
D (Local) vs. B (Global)	1.14	1.40

^†^ *p* < .1

* *p* < .05

** *p* < .01. Significance was calculated using an approximate t-test.

Our results show that transformational leadership values at the glocal identity type corner (Point A) and marginal identity type corner (Point C) were significantly higher than at the global identity type corner (Point B, *p* < .05). Between the glocal identity type corner and local identity type corner (Point D) this difference was marginally significant (*p* < .10). The difference between the marginal and local identity type corners was not significant.

The results for the leadership effectiveness criterion were similar for the differences between the glocal and global identity type corners (*p* < .01), and between the marginal and global identity corners (*p* < .01). Marginally significant differences were found between the glocal and local identity type corners (p < .10) and between the marginal and local identity corners (p < .10). There was no significant difference between the values of the balanced identity corners (glocal and marginal identity types), or between the unbalanced identity corners (global and local identity types), for either transformational leadership or leadership effectiveness.

Next, we tested the curvatures of the four interest lines that form the edges of the response surface (see Table 4) and that connect the unbalanced identity corners with the balanced identity corners (lines B–A, D–A, C–B, D–C), and the curvatures of the two diagonal lines (fit line: A–C; misfit line: B–D). The curvatures were significantly negative for the lines between the local and marginal identity type corners (line D–C) and between the global and glocal identity corners (line B–A), indicating that the surface curves downward in a concave manner for both transformational leadership (B = -.11, p < .05) and perceived leadership effectiveness (B = -.12, p < .05).

**Table 4:** Curvatures of all interest lines.

	Transformational leadership	Leadership effectiveness
Along the edges of the surface		
Glocal (A)–Global (B)	-0.11* (0.05)	-0.12* (0.06)
Glocal (A)–Local (D)	0.08 (0.06)	0.06 (0.08)
Marginal (C)–Global (B)	0.08 (0.06)	0.06 (0.08)
Marginal (C)–Local (D)	-0.11* (0.05)	-0.12* (0.06)
Along diagonal lines		
Marginal (C)–Glocal (A)	0.13 (0.08)	0.16 (0.10)
Local (D)–Global (B)	-0.21 (0.14)	-0.28 (0.18)

* p < .05.

**Fig 5:**
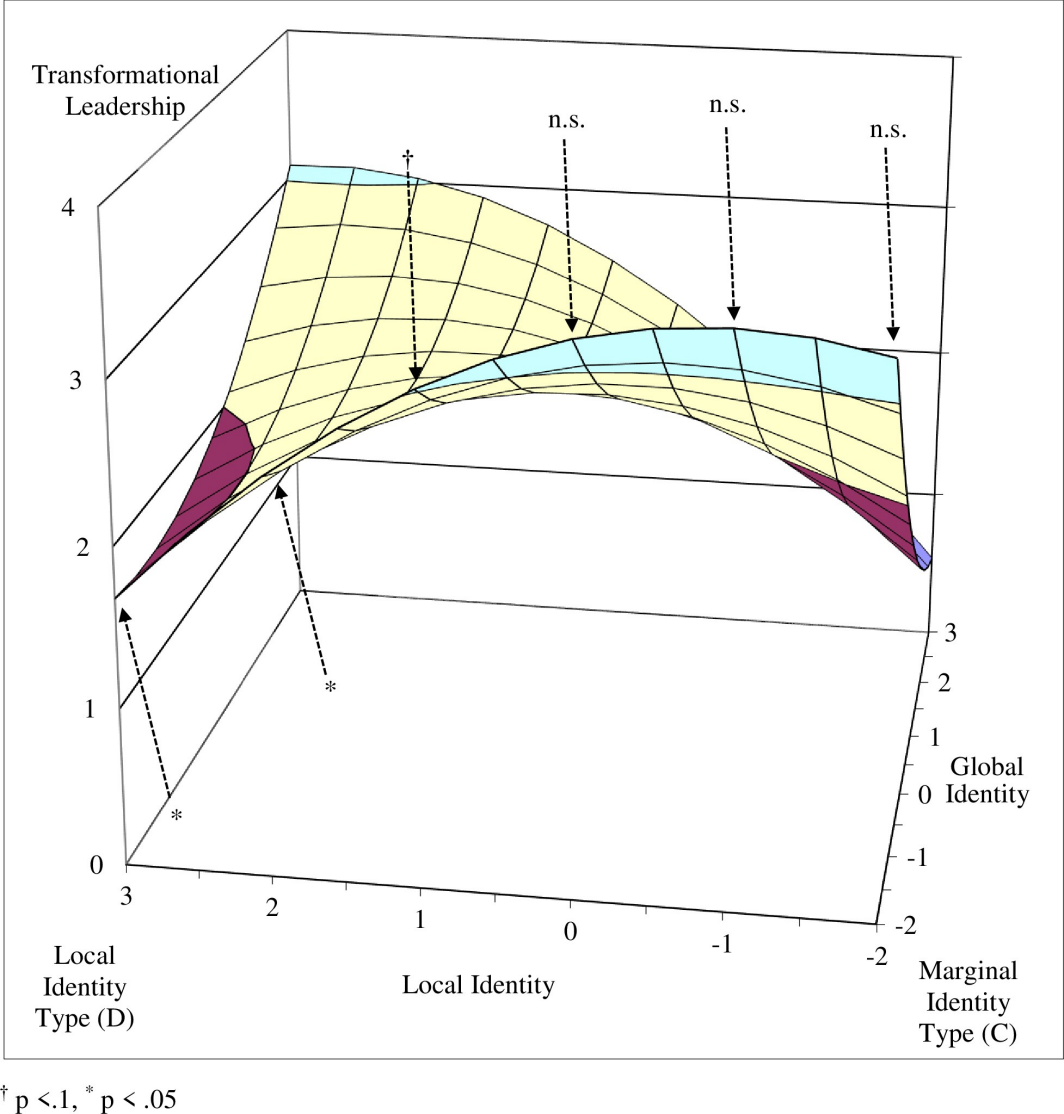
Tangent slope significance along the local–marginal line as predictor of transformational leadership.

**Fig 6:**
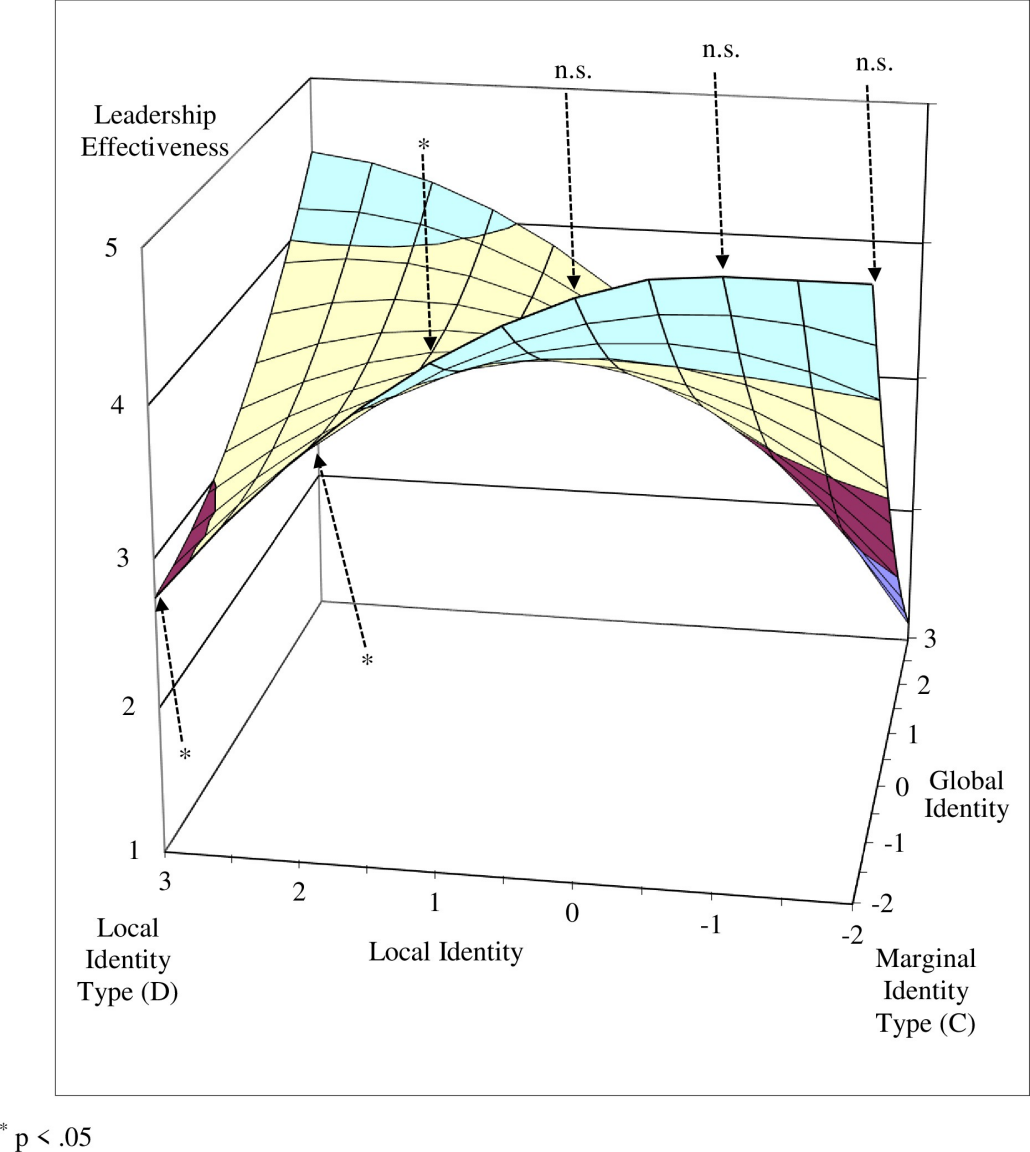
Tangent slope significance along the local–marginal line as predictor of leadership effectiveness.

Since the curvatures of these two lines were significant, we further tested the tangent slopes at particular points (see Table 5). We found that the tangent slopes at the interest line between the local and marginal identity type corners (where global identity is fixed at a value of -2) were not significant for either transformational leadership or leadership effectiveness when X (local identity) values were equal to -2, -1, and 0. However, the tangent slopes were marginally significant for transformational leadership and significant for leadership effectiveness when local identity was equal to 1, and were significant for both when local identity was equal to 2 and 3 (see Figs 5 and 6, and Table 5). These lines, which take a concave shape, indicate that when the discrepancy (i.e., unbalance) between global and local identity is low, there is no significant change in the level of transformational leadership and leadership effectiveness. However, when the discrepancy is high (global identity is equal to -2 and local identity is equal to 1, 2, or 3), a decrease is observed in both transformational leadership and leadership effectiveness.

**Table 5:** Wald tests for slopes of tangents along edges of response surfaces.

Corresponding point on response surface	When Y (global identity) = –2 C(Marginal) D(Local)						When Y (global identity) = 3 B(Global) A(Glocal)					
Local identity (x)	X = -2	X = -1	X = 0	X = 1	X = 2	X = 3	X = -2	X = -1	X = 0	X = 1	X = 2	X = 3
Transformational leadership	.23	.01	-.22	-.45†	-.68*	-.91*	1.08 **	.85**	.62**	.39*	.17	-.06
Leadership effectiveness	.11	-.13	-.37	-.62*	-.85*	-1.10*	1.22**	.99**	.74**	.50*	.26	.02

^†^ p < .1

*p < .05

** p < .01.

**Fig 7:**
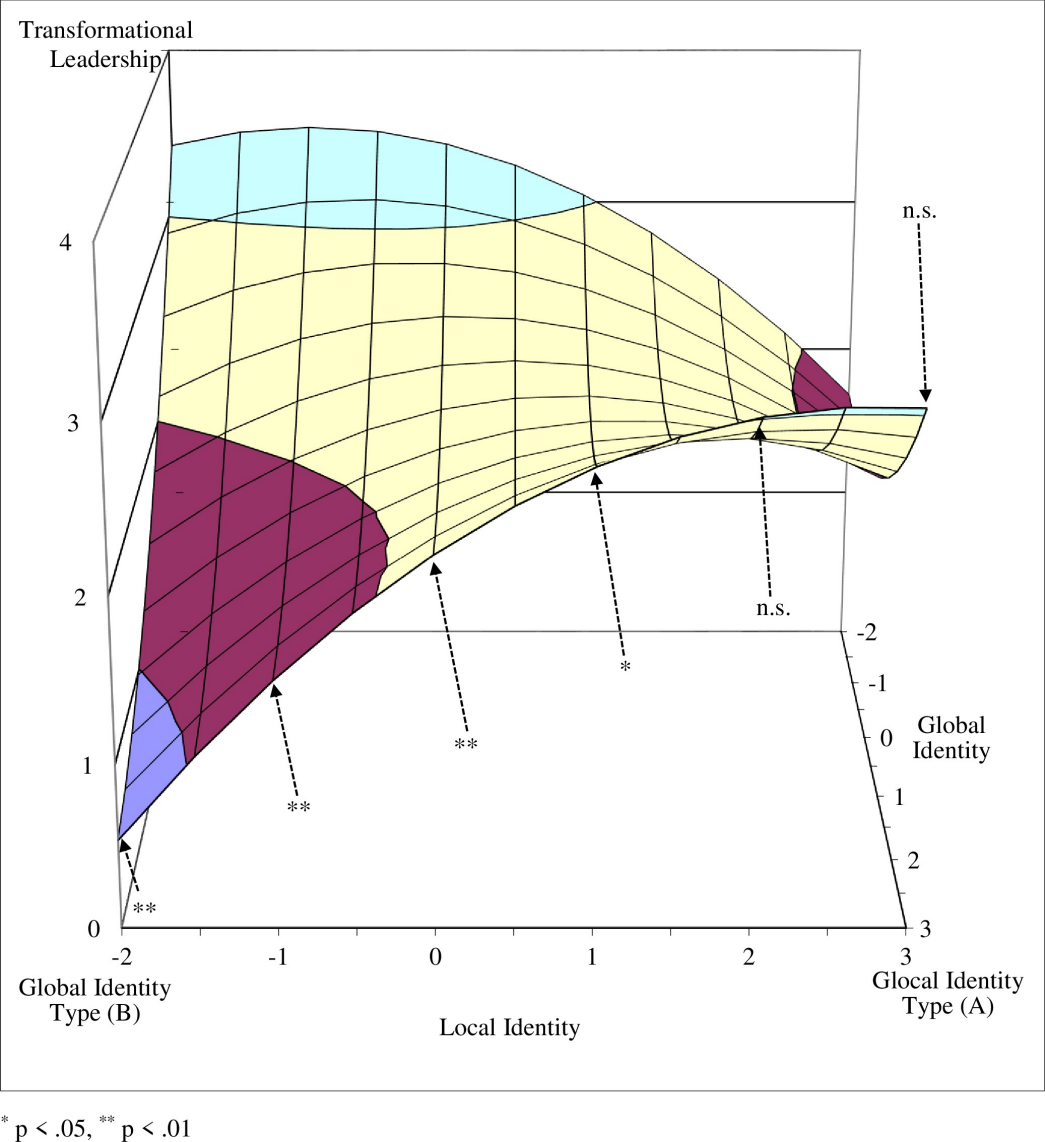
Tangent slope significance along the global–glocal line as predictor of transformational leadership.

**Fig 8:**
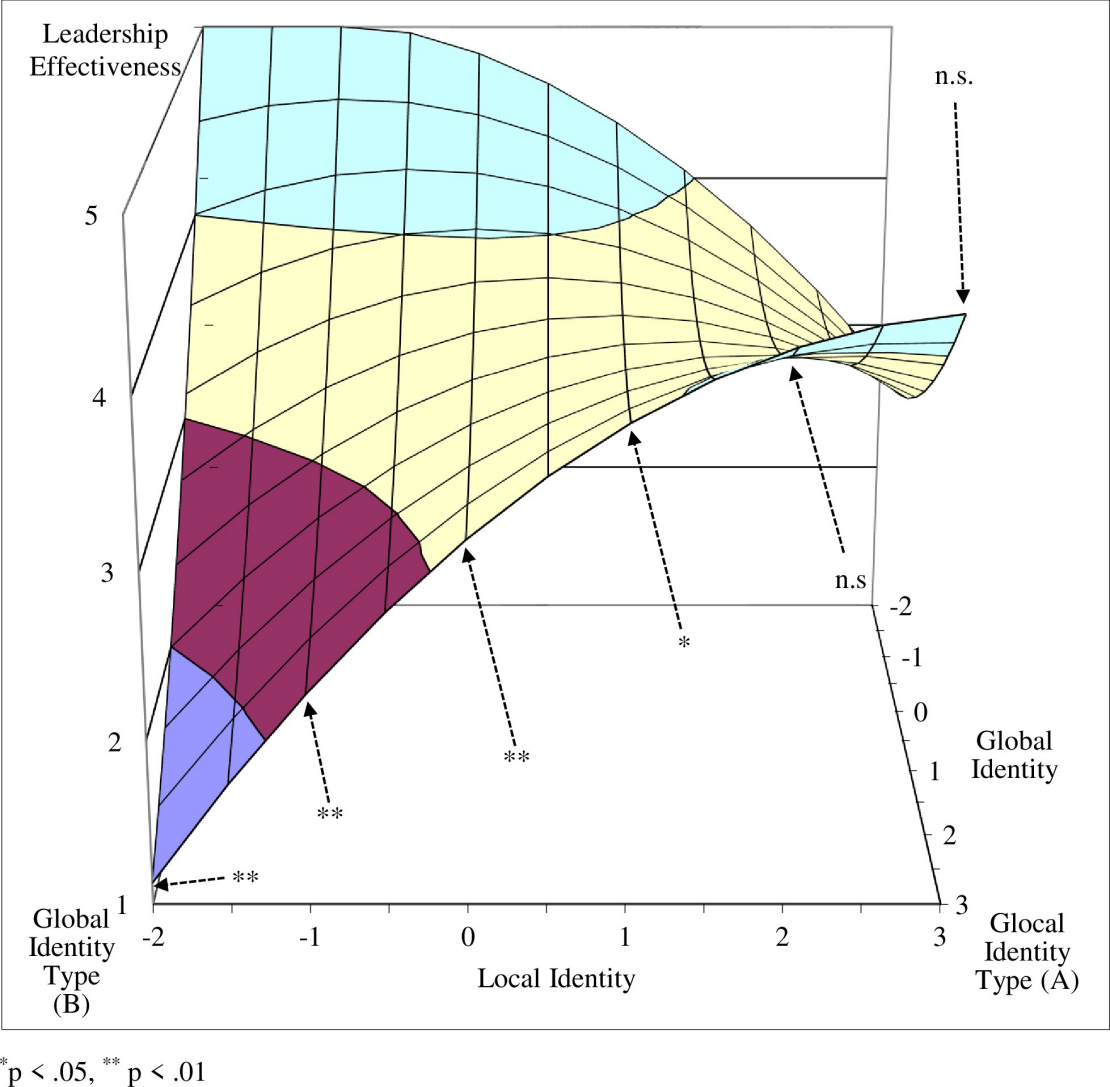
Tangent slope significance along the global–glocal line as predictor of leadership effectiveness.

A similar pattern was found for the line between the global and glocal identity corners (where global identity is fixed at a value of 3). In both cases, the tangent slopes were not significant when the discrepancy was low (i.e., the local identity (X) value was equal to 3 or 2), but were significant at higher discrepancies, where local identity (X) was equal to 1, 0, -1 or -2 (see Figs 7 and 8, Table 5). These results support Hypotheses 1 and 2.

The curvatures of the interest lines along the edge of the surface between the marginal and global identity type corners (line C–B) and between the local and glocal identity type corners (line D–A) were not significant for either transformational leadership (B = .08, n.s.) or leadership effectiveness (B = .06, n.s.). The tangent slope of the former line, however, was significant when global identity was equal to zero for both transformational leadership (B = -0.65, p < .05) and leadership effectiveness (B = -0.83, p < .01). This suggests that both transformational leadership and leadership effectiveness decrease along the line between the (balanced) marginal identity corner and the (unbalanced) global identity corner, further supporting Hypotheses 1 and 2. The tangent slope at the same point was not significant for the line between the local and glocal identity type corners for either transformational leadership (B = 0.20, n.s.) or leadership effectiveness (B = 0.29, n.s.).

To test the mediating effect of transformational leadership on the relationship between the joint effect of leader’s global and local identities and leadership effectiveness (H3), we ran a mediation model. As shown in Table 2 (leadership effectiveness model, Step 2), the five polynomial terms served to predict leadership effectiveness. In Step 3 we added transformational leadership into the regression, and found that result to be significant (0.70, *p* < .01). The standardized path coefficient of the joint effect of the global and local identities of leaders on transformational leadership was β = .31 (*p <* .01), and the path coefficient for the relationship between transformational leadership and leadership effectiveness was β = .64 (*p* < .01). We calculated the indirect effect by multiplying the coefficients of these paths and found that the indirect effect was significant (β = .20, 95% CI [.10, .31]). The results show that transformational leadership mediates the joint effects of leaders’ global and local identities on leadership effectiveness, thus supporting Hypothesis 3.

Finally, although we did not propose hypotheses regarding the relationships between the two balanced and two unbalanced identity types, we explored both the curvatures and slopes along the diagonal lines between the balanced identity type corners (marginal and glocal: C–A), and between the unbalanced identity type corners (global and local: B–D). None of these were found to be significant for either transformational leadership or leadership effectiveness.

## Discussion

In light of the growing presence of multicultural teams in global organizations, it has become essential to advance our understanding of how to enhance their leaders’ effectiveness [31]. This study explored how leaders’ social identities are related to transformational leadership behaviors and consequently to leadership effectiveness in this context. Specifically, we proposed a model that explains how particular socio-cultural identity configurations promote transformational leadership behaviors in leaders of multicultural teams, and thereby enhance their effectiveness.

Our findings show that leaders with balanced identity configurations, both glocal and marginal, demonstrated more transformational leadership behaviors and were perceived by team members as more effective than leaders with unbalanced local or global identity configurations. While the results for transformational leadership behaviors were less convincing when comparing identity configuration scores, the relative interest lines revealed patterns indicating that the greater the discrepancy between global identity and local identity values, the greater the decline in transformational leadership behaviors and leadership effectiveness. Such patterns further support our argument that the balance between the global and local identities of multicultural team leaders drives an increase in transformational leadership behaviors, and consequently improves their effectiveness.

### Theoretical contributions

This study advances the current understanding of how leaders’ identities are related to transformational leadership and effectiveness in a multicultural team context. Integrating ideas from the global acculturation model and social identity complexity theory, we show that balance between the leader’s global and local identities, is related to transformational leadership in a multicultural environment. These findings support the claim that balanced cultural identity configurations are associated with the sort of unbiased cultural perspective [20,33] needed to relate to followers from different cultural backgrounds individually, while at the same time instilling a sense of common purpose and dedication to the team’s tasks. They also support theoretical claims in the leadership literature that see self-identity as a driving force of charismatic leadership behaviors such as transformational leadership [26–29,108]. More broadly, the findings help delineate how leaders’ multiple social identities may interact to explain complex behaviors; and they underscore the value of considering these identities in terms of their relative strengths and the balance between them, rather than simply examining each one individually. In this respect, our approach adds to previous theories and findings which focus solely on the global identity, and which suggest that high global identity itself allows individuals from diverse backgrounds to be successful and effective in the global community [12,24]. These previous theoretical perspectives rely on the logic that groups perform better to the extent that their members learn to think of themselves as belonging to a common ingroup, and suggest that leaders with a strong (vs. weak) global identity will be more effective in multicultural teams because they are better able to communicate what the members have in common. We rework this logic by arguing that a balance between a global and local identity gives leaders the tools not only to communicate what is shared by the group, but also to both respect and capitalize on members’ differences through transformational leadership. Our approach is also in line with calls for greater exploration of how identity configurations influence and interact with complex behaviors [109,110].

Our findings also contribute to the acculturation literature, by addressing the theoretical tension between traditional acculturation logic and past findings on marginals’ effectiveness in the multicultural context. Whereas traditional acculturation theory suggests that strong identification with one cultural group is better than low identification with two [21], this logic fails to explain findings that show marginals may adapt to a multicultural context more successfully than individuals who identify strongly with one culture [23,61]. It may be that traditional acculturation models are more relevant for immigrants’ acculturation to a host country, where individuals are asked to adapt to a move from one local culture to another. The present study, by contrast, concerns acculturation in a global multicultural setting, where there is no expectation that the local culture must be subsumed or left behind. In this case, our revised logic argues that balance between the local and global identities is key to successful adaptation [33].

In this vein, our perspective is focused on the commonalities between the marginal and glocal configurations. However, we cannot ignore the differences between them. In particular, individuals with glocal and marginal configurations may have similar cultural knowledge of both groups, but only the former have high levels of identification [64,111]. Nevertheless, our view and findings suggest that although the emotional attachment of glocal and marginal configurations differ, both identities configurations can be effective. Leaders with a glocal configuration who identify strongly with both cultural groups may enjoy more identification related motivational advantages and show concern for these groups’ welfare. Leaders with marginal configuration do not possess this motivational advantage related to their identification with cultural groups. This might be a weakness, as leaders with low attachment to the global group can have weak social ties [21,22] and consequently low commitment to the multicultural team. On the other hand, low emotional attachment can be an advantage for effective global leaders, enabling them to avoid manifesting biased behaviors triggered by cultural stereotypes, to understand the importance of these cultures for their followers, and to use cultures’ positive aspects to promote their goals [33]. Indeed, scholars have suggested that greater autonomy and cultural independence are advantages of a marginal configuration [23,61]. These points complement earlier suggestions that marginal leaders, as cultural group members with a low emotional attachment to these cultures, can “pick and choose what they deem to be appropriate from each culture, rather than allowing society to dictate ascribed expectations” [20, p. 537]. Thus, leaders with the two different types of balanced configurations may have some advantages in common, namely cultural knowledge and an unbiased perspective, alongside different advantages and weaknesses.

### Managerial implications

Our results suggest that organizations which operate internationally, and which rely on culturally diverse work teams, should encourage development of a balanced identity when training team leaders. First, organizations should encourage leaders to recognize the existence of both national–local and global elements in the cultures of the group and its members. While exposure to a multicultural team context is likely by itself to encourage identification with the global culture [55], leaders may benefit from cross-cultural training to improve their sensitivity to local cultures (e.g., [112]). Second, leaders should be trained to harness members’ cultural diversity to meet the team’s goals while facilitating a sense of cohesiveness and belonging (e.g., [74,113]). This leadership training should further focus on teaching participants how to connect this cultural perspective to transformational leadership behaviors in ways that will improve proactivity among culturally diverse team members.

### Limitations and future research

Our study offers insight into the relationship between the social identity configurations of leaders, their behaviors, and their effectiveness in the context of multicultural teams. These results constitute a foundation for future studies that will continue this line of research and overcome the study’s limitations.

In this study, we focus on transformational leadership behaviors to examine how leaders’ identities influence their effectiveness. We chose to spotlight transformational leadership because it facilitates positive team processes such as creativity, communication, and trust [76,114,115], and because it has been shown to have effects on performance beyond transactional leadership behaviors [42,49,116]. However, leaders’ identity configurations may also promote other effective leadership behaviors in the multicultural team context. Future studies may broaden the exploration of relationships between identity configurations and other leadership behaviors, either context-related or more general.

Another factor that requires consideration is the virtual context of our study. Although many multicultural teams in global organizations are virtual, some are co-located. The fact that virtual teams rely on technology to communicate [2] can influence the relationships between team members. For example, in one study, cultural diversity tended to be associated with more conflict and less social integration when teams were co-located compared to when they were geographically dispersed [5], and creation of shared identity may be especially challenging when co-located teams include leaders and members living in host countries, who may have three salient identities (home, host, and global) [23].On the other hand, the limited scope for social cues in virtual teams may make it more difficult for virtual team leaders to demonstrate transformational leadership behaviors [117]. These factors can affect both the impact of leaders’ identity configurations on their behavior, and the impact of leadership behaviors on team and leadership effectiveness. Therefore, we recommend testing our model, or similar models, in co-located multicultural teams.

A further concern relates to the external validity of the setting, given that our participants were MBA students. However, four factors mitigate this concern. First, most of our participants were part-time students who worked part-time or full-time in global or local organizations; and most (75%) reported that they had worked in multicultural teams in the past. Hence, the profile of these students is similar to that of educated employees in many organizations. Second, research reveals a high correlation between the effect sizes of field studies and non-field study settings [118]. Third, our participants communicated with each other intensively, on a daily basis, throughout the entire month, simulating fieldwork. Finally, the features of our research setting are typical for many multicultural teams in the workplace. For instance, in a recent survey of a consulting group, 48% of the respondents had never met other team members in person, suggesting that the virtual work context is an increasing trend [119].

An additional limitation stems from the research design. Due to the project timeline, we measured team members’ reports of both transformational leadership behaviors and leadership effectiveness only at the end of the project. Although this is not ideal, our CFA analyses confirmed that team members’ perceptions of transformational leadership and leadership effectiveness were separate constructs, and that common method variance did not appear to bias the relationships between these variables. Moreover, some scholars have argued that followers develop their perceptions of leadership effectiveness based on their perceptions of their leaders’ behaviors [9,10,16], providing theoretical support for our research model.

Finally, we explored how leadership identity configurations are related to leadership effectiveness in multicultural teams. An interesting question that remains open is how leadership identity configurations are related to leadership emergence in this context. Recent research findings indicate that high global identity is positively related to multicultural team leadership emergence [23,30]. During team development, leaders with a high global identity may provide signals that fit followers’ expectations for prototypical leadership in this context [120,121]. More research is needed to explore further whether balanced configurations are related to this process. Moreover, leadership effectiveness results from actual leadership behaviors that require leader–follower interactions over time. In this context, it is likely that some emergent leaders who seem to be a good fit for the role in the early stages of the team’s lifecycle will not be effective leaders later on. We encourage scholars to explore this direction in future longitudinal studies.

## Conclusion

Leaders play an essential role in leveraging the benefits and mitigating the challenges of multicultural teams. Transformational leadership is thought to be effective across organizational contexts, yet we know little about the antecedents of transformational leadership behaviors and effectiveness in multicultural settings. By incorporating global acculturation and identity complexity principles into the multicultural team context, this study delineates how balance between leaders’ global and local identities leads to transformational leadership behaviors and leadership effectiveness. This study contributes to research on identities as a driving force of transformational leadership behaviors, and to the emergent literature on global acculturation.

## Supporting information

S1 Appendix Common Method Variance (CMV) analysis. (DOCX)

S2 Appendix Testing curvatures along the edge of a response surface. (DOCX)

S1 File List of measures. (DOCX)
