# Correction: Global and local identities on the balance scale: Predicting transformational leadership and effectiveness in multicultural teams

**DOI:** 10.1371/journal.pone.0258025

**Published:** 2021-09-23

**Authors:** Alon Lisak, Raveh Harush

Fig 2 is incorrect. The authors have provided a corrected version here.

**Fig 2:**
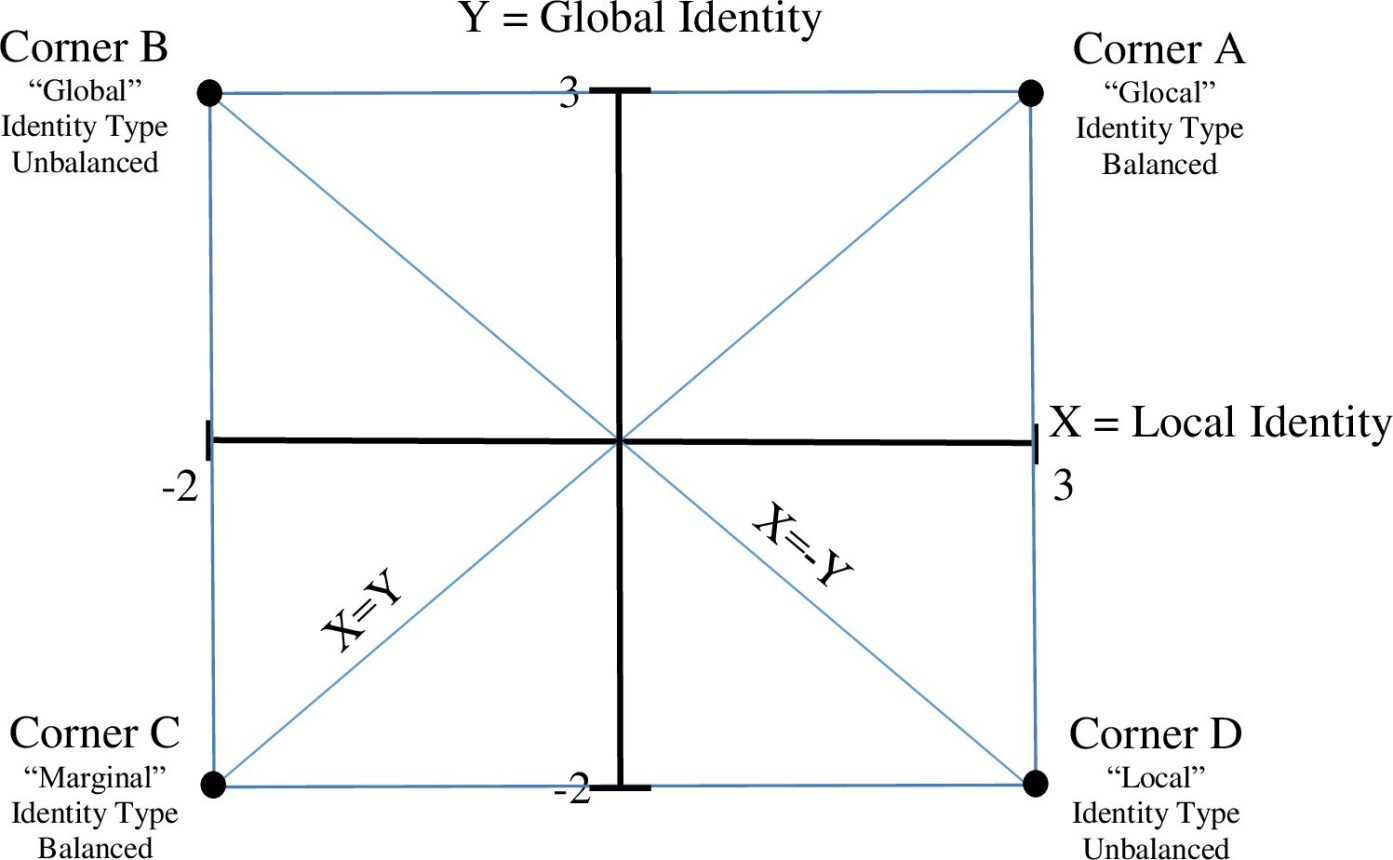
Schematic description of lines of interest between the four identity types.
