# Supplementary material for: Global and local identities on the balance scale: Predicting transformational leadership and effectiveness in multicultural teams
Source: PLoS One. 2021 Jul 14;16(7):e0254656. doi: 10.1371/journal.pone.0254656 (PMC8279353; doi:10.1371/journal.pone.0254656)
Supplement: S1 Appendix — (DOCX) [file pone.0254656.s001.docx]

**Appendix A**

**Common Method Variance (CMV) Analysis**

Procedure:

Since the data were clustered, we used the TYPE=COMPLEX command to control the within-cluster correlation (causing Mplus to use a sandwich estimator). First, we tested a CFA model with the marker variable. The CFA marker model included three factors: social desirability [1], transformational leadership [2], and leadership effectiveness [3]. To preserve model power, we use the aggregated scores of the three transformational leadership subscales as indicators (individualized consideration, intellectual stimulation, and charisma [inspirational motivation + idealized influence]). Next, following the procedure of [4, 5], we tested a baseline model where the two correlations between the marker and substantive latent variables were set to zero, and the unstandardized regression weights and variances for the marker variable were fixed to the values obtained from the CFA marker model. Third, we tested a constrained model (Model-C) where the nine-factor loadings from the latent marker variable were constrained to be equal. Fourth, we tested an unconstrained model (Model-U) where the nine-factor loadings from the latent marker variable were freely estimated. Finally, we tested a restricted model (Model-R), where the substantive factor covariances from Model-U were set to their values from the baseline model. The results are shown in Table 6. The constrained model (Model-C) offered a marginally significantly better fit than the baseline model (Δχ^2^ (2.77, 1), p<.1), which indicates that there was marginal shared CMV between the indicators of the substantive variables and the latent marker variable. When compared with the constrained model, the unconstrained model (Model-U) fit the data better (Δχ^2^ (23.35, 8), p<.01), which indicates that CMV was not constant for all indicators. The restricted model (Model-R) was not significantly different from Model-U (Δχ2 (0.00, 1), ns), which indicates that the presence of CMV did not appear to bias the relationships among the substantive variables (see [4]).

**Table 6**

CMV- Fit indices and comparison between models

| Model | χ^2^ | | SCF | CFI | TLI | SRMR | Δχ^2^ | | | Model comparison |
| --- | --- | --- | --- | --- | --- | --- | --- | --- | --- | --- |
|  | Value | df |  |  |  |  | Trd | Δdf | p |  |
| CFA with marker Variable | 216.531 | 74 | 1.0752 | .93 | .90 | .051 |  |  |  |  |
| Baseline | 222.018 | 86 | 1.0686 | .93 | .93 | .066 |  |  |  |  |
| Model-C | 219.185 | 85 | 1.0643 | .93 | .93 | .051 | 2.77 | 1 | <.1 | Vs. Baseline |
| Model-U | 195.174 | 77 | 1.0406 | .94 | .93 | .047 | 23.35 | 8 | <.01 | Vs. Model-C |
| Model-R | 192.964 | 78 | 1.0525 | .94 | .93 | .047 | 0.00 | 1 | ns | Vs. Model-U |

Note: N=221, χ^2^ = Satorra-Bentler scaled chi-square; SCF= scaling correction factor for MLR; CFI= comparative fit index; TLI=  Tucker-Lewis index; SRMR= standardized root mean squared residual; TRd = Satorra-Bentler scaled chi-square difference test; cd: difference test scaling correction;  C = constrained; U = unconstrained; R = restricted.   Δχ^2^ (Trd,cd) = chi-square difference test. Scaled chi-square cannot be used for a chi-square difference test of nested models because a difference between two scaled chi-squares for nested models is not distributed as a chi-square. Therefore, we used the Sattora-Bentler scaled chi-square procedure [6] in order to compute a chi-square difference test between the nested models.

The chi-square difference test procedure was as follows: T0/1 =  Satorra-Bentler scaled chi-square score, Model 0/Model 1; D0/1= degree of freedom, Model 0/Model 1; C0/1 =  scaling correction factor for MLR, Model 0/ Model 1. Step 1: Difference test scaling correction (cd) = (d0 * c0 - d1*c1)/(d0 - d1). Step 2:  Satorra-Bentler scaled chi-square difference test (TRd)= (T0*c0 - T1*c1)/cd.

Example: Comparison between the constrained and unconstrained models. Model 0 – Constrained: T0=219.185; d0= 85; c0=1.0643; Model 1 – Unconstrained: T1= 195.174; d1= 77; c1= 1.0406. cd= (85*1.0643-77*1.0406)/85-77= 90.4655- 80.1262/8= 1.29. (TRd)= (T0*c0 - T1*c1)/cd  (219.185*1.0643- 195.174*1.0406)/1.2924125=23.35.  χ^2^ (TRd, d0-d1)= 2(23.35, 8), p<.01.

**References**

1. Paulhus DL. Balanced inventory of desirable responding (BIDR). Accept Commitment Ther Meas Package. 1988: 41–43.
2. Avolio BJ, Bass BM. Individual consideration viewed at multiple levels of analysis: A multi-level framework for examining the diffusion of transformational leadership. Leadersh Q. 1995;6(2): 199–218. doi:[10.1016/1048-9843(95)90035-7](https://doi.org/10.1016/1048-9843(95)90035-7).
3. Denison DR, Hooijberg R, Quinn RE. Paradox and performance: Toward a theory of behavioral complexity in managerial leadership. Organ Sci. 1995;6(5): 524–540. doi:[10.1287/orsc.6.5.524](https://doi.org/10.1287/orsc.6.5.524).
4. Williams LJ, Hartman N, Cavazotte F. Method variance and marker variables: A review and comprehensive CFA marker technique. Organ Res Methods. 2010 Jul;13(3): 477–514.
5. Shuck B, Nimon K, Zigarmi D. Untangling the predictive nomological validity of employee engagement: Partitioning variance in employee engagement using job attitude measures. Group Organ Manag. 2017 Feb;42(1): 79–112. doi:[10.1177/1059601116642364](https://doi.org/10.1177/1059601116642364).
6. Satorra A, Bentler PM. A scaled difference chi-square test statistic for moment structure analysis. Psychometrika. 2001 Dec 1;66(4): 507–514. doi:[10.1007/BF02296192](https://doi.org/10.1007/BF02296192).
