# Supplementary material for: Global and local identities on the balance scale: Predicting transformational leadership and effectiveness in multicultural teams
Source: PLoS One. 2021 Jul 14;16(7):e0254656. doi: 10.1371/journal.pone.0254656 (PMC8279353; doi:10.1371/journal.pone.0254656)
Supplement: S2 Appendix — (DOCX) [file pone.0254656.s002.docx]

**Appendix B**

**Testing Curvatures Along the Edge of a Response Surface**

Polynomial regression analysis [1–3] involves estimating a combined linear and quadratic regression model, represented by Equation 1:

(1) $Z=b_{0}+ b_{1}X+b_{2}Y+b_{3}X^{2}+b_{4}XY+b_{5}Y^{2}+e.$

In our study, X and Y are global identity and local identity, respectively; Z is transformational leadership behaviors/ leadership effectiveness; b_1_ and b_2_ are the regression coefficients for global identity and local identity, respectively; b_3_ is the regression coefficient for global identity squared; b_4_ is the regression coefficient for the interaction between global and local identities; and b_5_ is the regression coefficient for local identity squared.

To test the curvatures of the four lines of interest along the edge of the response surface, we relied on the work presented by Cohen et al. [4] and Lee and Antonakis [5] and developed two equations.

Fig 2 demonstrates that the line between corners A (glocal identity type) and D (local identity type) represents subjects for whom the global identity value (X) is allowed to fluctuate freely in the defined range (from (-2) to 3), whereas the local identity (Y) value is fixed to Y* (Y* = 3 for this specific line, at the highest level of the local identity continuum). Hence, for this line, we replace Y with Y* in Equation 1. The resulting equation is:

(2) $Z=\left( b_{0}+{Y^{*}*b}_{2}+{\left( Y^{*} \right)^{2}*b}_{5} \right)+ \left( b_{1}+Y^{*}*b_{4} \right)X+b_{3}X^{2}+e.$

The curvature along this line (where is X allowed to fluctuate) equals $b_{3}.$ Please note that the curvature is fixed and equals $b_{3}$ for any choice of $Y^{*}$.

The pattern for the mirror line, which connects corner B (global identity type) and corner C (marginal identity type), is the same. X (global identity) is allowed to fluctuate freely as for the previous line, and the local identity (Y) value is fixed to Y* (specifically for this line, Y* equals -2). Since the curvature does not depend on the specific Y*, as explained for the line above, the curvature of this line will also equal$b_{3}$.

The line between corners A (glocal identity type) and B (global identity type) represents subjects for whom the global identity (*X*) value is fixed to X* (X* = 3 for this specific line), whereas the local identity value (*Y*) is allowed to fluctuate freely in the defined range (from (-2) to 3). For this line, we replace *X* with X* in Equation 1 to obtain:

$$(3) Z=\left( b_{0}+ X^{*}*b_{1}+\left( X^{*} \right)^{2}*b_{3} \right)+\left( b_{2}+{X^{*}*b}_{4} \right)Y+b_{5}Y^{2}+e.$$

Hence, the curvature along this line (where Y is allowed to fluctuate) equals $b_{5}$. Again, please note that the curvature for this line equals $b_{5}$ for any choice of $X^{*}$.

Since the pattern of the mirror line between corner D (local identity type) and corner C (marginal identity type) is similar (i.e., the global identity (*X*) value is fixed to X* (X* = -2 for this specific line)), and the local identity value (*Y*) is allowed to fluctuate freely in the defined range (from (-2) to 3), this curvature also equals $b_{5.}$

**References**

1. Edwards JR. The study of congruence in organizational behavior research: Critique and a proposed alternative. Organ Behav Hum Decis Process. 1994;58(1): 51–100. doi:[10.1006/obhd.1994.1029](https://doi.org/10.1006/obhd.1994.1029).
2. Edwards JR. Alternatives to difference scores as dependent variables in the study of congruence in organizational research. Organ Behav Hum Decis Process. 1995;64(3): 307–324.
3. Edwards JR, Parry ME. On the use of polynomial regression equations as an alternative to difference scores in organizational research. Acad Manag J. 1993;36(6): 1577–1613.
4. Cohen A, Nahum-Shani I, Doveh E. Further insight and additional inference methods for polynomial regression applied to the analysis of congruence. Multivariate Behav Res. 2010;45(5): 828–852. doi:[10.1080/00273171.2010.519272](https://doi.org/10.1080/00273171.2010.519272), PubMed: [21103324](https://www.ncbi.nlm.nih.gov/pubmed/21103324).
5. Lee YT, Antonakis J. When preference is not satisfied but the individual is: How power distance moderates person–job fit. J Manag. 2014;40(3): 641–675.
