# Supplementary material for: Global and local identities on the balance scale: Predicting transformational leadership and effectiveness in multicultural teams
Source: PLoS One. 2021 Jul 14;16(7):e0254656. doi: 10.1371/journal.pone.0254656 (PMC8279353; doi:10.1371/journal.pone.0254656)
Supplement: S1 File — (DOCX) [file pone.0254656.s003.docx]

**List of Measures**

**Leaders**

**Local and global identity scales (Shokef & Erez, 2006;2008)**

**Local Identity**

1. I see myself as part of my society (e.g., Israeli, American, Korean, etc.)
2. I feel a strong attachment towards the society, I belong to.
3. I define my self as an ____ (your nationality- e.g., Israeli, American, Korean, etc.)
4. I relate to people from my country as if they were close acquaintances/associates
5. I feel a strong attachment towards people from my country

**Global Identity**

1. I see myself as part of the global international community
2. I feel a strong attachment towards the world environment I belong to
3. I would define myself as a citizen of the global world
4. I relate to people from other parts of the world as if they were close acquaintances/associates
5. I feel a strong attachment towards people from all around the world

**General Self Efficacy (Chen, Gully & Eden , 2001).**

1. I will be able to achieve most of the goals that I have set for myself.
2. When facing difficult tasks, I am certain that I will accomplish them.
3. In general, I think that I can obtain outcomes that are important to me.
4. I believe I can succeed at most any endeavor to which I set my mind.
5. I will be able to successfully overcome many challenges.
6. I am confident that I can perform effectively on many different tasks.
7. Compared to other people, I can do most tasks very well.
8. Even when things are tough, I can perform quite well.

| 1. I believe in the importance of art. |
| --- |
| 1. I have a vivid imagination. |
| 1. I tend to vote for liberal political candidates. |
| 1. I carry the conversation to a higher level. |
| 1. I enjoy hearing new ideas. |

**Openness to experiences (Buchanan, Johnson & Goldberg, 2004)**

Gender: Male / Female

Age:________ years

How many languages do you speak?

**Members**

**Tranformational Leadership (Avolio & Bass, 2004).**

**(My team leader is….)**

**Individualized Consideration**

Spends time teaching and coaching

Treats me as an individual rather than just as a member of a group

Considers me as having different needs, abilities, and aspirations from others

Helps me to develop my strengths

**Intellectual Stimulation**

Re-examines critical assumptions to question whether they are appropriate

Seeks differing perspectives when solving problems

Gets me to look at problems from many different angles

Suggests new ways of looking at how to complete assignments

**Idealized Influences (Behavior)**

Talks about their most important values and beliefs

Specifies the importance of having a strong sense of purpose

Considers the moral and ethical consequences of decisions

Emphasizes the importance of having a collective sense of mission

**Inspirational Motivation**

Talks optimistically about the future

Talks enthusiastically about what needs to be accomplished

Articulates a compelling vision of the future

Expresses confidence that goals will be achieved

**Leadership effectiveness (Based on Denison, Hooijberg and Quinn, 1995)**

The team leader meat managerial performance standards during the project

The team leader's performance met my expectations during the project

The team leader facilitated the accomplishment of the team mission during the project

The team leader was a role model during the project

The team leader succeeded in his/her role during the project.

The team leader was effective during the project

Gender: Male / Female

Age:________ years

How many languages do you speak?

**Local and global identity scales (Shokef & Erez, 2006;2008)**

**Local Identity**

I see myself as part of my society (e.g., Israeli, American, Korean, etc.)

I feel a strong attachment towards the society, I belong to.

I define my self as an ____ (your nationality- e.g., Israeli, American, Korean, etc.)

I relate to people from my country as if they were close acquaintances/associates

I feel a strong attachment towards people from my country

**Global Identity**

I see myself as part of the global international community

I feel a strong attachment towards the world environment I belong to

I would define myself as a citizen of the global world

I relate to people from other parts of the world as if they were close acquaintances/associates

I feel a strong attachment towards people from all around the world

**Social Desirebility (Paulhus, 1988)**

I don’t care to know what others people really think of me

I always know why I like things

Once I’ve made up my mind, other people can seldom change my opinion

I never regret my decisions

It is alright with me if some people happen to dislike me
